# Data in support of protocol for rat single muscle-fiber isolation and culture

**DOI:** 10.1016/j.dib.2015.04.016

**Published:** 2015-05-02

**Authors:** Yusuke Komiya, Judy E. Anderson, Mariko Akahoshi, Mako Nakamura, Ryuichi Tatsumi, Yoshihide Ikeuchi, Wataru Mizunoya

**Affiliations:** a Department of Bioresource Sciences, Faculty of Agriculture, Kyushu University, Higashi-ku, Fukuoka 812-8581, Japan; b Department of Biological Sciences, University of Manitoba, Winnipeg, Canada R3T 2N2

**Keywords:** Skeletal muscle, Muscle fiber type, Primary culture, Myotube, Western blotting

## Abstract

This data article contains data related to the research article entitled, "Protocol for rat single muscle-fiber isolation and culture" by Komiya et al. [1]. It has yet to be shown whether adult myosin heavy chain (MyHC) isoforms are expressed at a readily detectable level in cultured myotubes. In this study, we examined whether adult MyHC isoforms could be detected in myotubes differentiated from rat satellite cells using a Western blotting assay and specific antibodies against slow MyHC, fast MyHC and pan-MyHC. Results showed that slow adult MyHC isoforms were faintly detected in myotubes, suggesting that rat myotubes express adult MyHC isoforms although that amount is very low.

**Specifications table**

**Table:** 

Subject area	Biology
More specific subject area	Skeletal muscle biology, cell physiology
Type of data	Image
How data was acquired	Western blotting
Data format	Raw
Experimental factors	Muscle satellite cells isolated from rats were cultured for 6 days to form myotubes and subjected to a whole cell protein extraction
Experimental features	The protein expression of adult slow MyHC isoforms was examined by Western blotting
Data source location	Fukuoka, Japan
Data accessibility	Data is supplied in this article

**Value of the data**

• The low but detectable amount of adult slow MyHC was confirmed in cultured myotubes differentiated from rat satellite cells for 6 days by Western blotting.
• The regulation of adult slow MyHC isoforms expression could be evaluated by Western blotting even though immature cultured myotubes.
• Undetectable level of adult fast MyHC isoforms appeared to be contained in myotubes in this experimental condition.

## 1 Data, experimental design, materials and methods

### 1.1 Sample preparation

Satellite cells were isolated from 4-week-old male Sprague–Dawley rats according to Allen et al. [2] with slight modification [3]. Briefly, muscles were excised, trimmed of fat and connective tissue, hand-minced with scissors, and digested for 1 h at 37 °C with 1.25 mg/ml protease type XIV (P5147; Sigma-Aldrich, USA). Cells were separated from muscle fiber fragments and tissue debris by differential centrifugation. In addition, to increase the purity of satellite cells in the cell mixture, cells were fractionated by Percoll density centrifugation according to Kastener et al. [4] with slight modification for a five-layered discontinuous density Percoll gradient (2 mL 27.5%–2 mL 35%–2 mL 40%–2 mL 55%–2 mL 90%). The fractionated cells (40–55% Percoll fraction) were plated at 2.0×10^4^ cells/cm^2^ on 6-well plates (4810-040; Iwaki brand Asahi Glass, Tokyo, Japan) coated with poly-l-lysine and fibronectin in alpha Modified Eagle׳s Minimum Essential Medium (α-MEM, Life Technologies, USA) containing 10% normal horse serum (HS, Invitrogen, USA), 1% antibiotic–antimycotic mixture (Life Technologies), and 0.5% gentamicin (Life Technologies). Cultures were placed in a humidified atmosphere of 5% CO_2_ at 37 °C. Forty-eight hours after plating, satellite cells were differentiated in 2% HS in Opti-MEM (Life Technologies) for 6 days to form myotubes. These myotubes were lysed with 200 μl/well SDS solution containing 10% SDS, 40 mM dithiothreitol (DTT), 5 mM EDTA, and 0.1 M Tris–HCl buffer at pH 8.0, followed by addition of Protease Inhibitor Cocktail for use with mammalian cell and tissue extracts (1:100, Nacalai Tesque, Japan). These cell lysates were diluted by an equal volume of 2×sample buffer (100 mM DTT, 4.0%w/v SDS, 0.16 M Tris–HCl (pH 6.8), 43% v/v glycerol, and 0.2% w/v bromophenol blue) and heated at 100 °C for 3 min. Animal experiments were performed according to the Guidelines for Animal Experiments of Kyushu University, and with the ethical approval of the Animal Care and Use Committee, Kyushu University (protocol #05-002-01)

### 1.2 Western blotting

Samples were loaded onto a 10% polyacrylamide gel, subjected to electrophoresis, and transferred to a Hybond PVDF membrane (GE Healthcare, USA) for 4 h at 38 V (constant voltage). Membranes were blocked with 5% skim milk diluted in Tris-buffered saline containing 0.1% Tween 20 (TBS-T) for 45 min at room temperature. The blots were then incubated overnight at 4 °C with gentle agitation in dilutions of primary antibodies, as follows: mouse monoclonal anti-fast MyHC (clone MY-32, Sigma-Aldrich, 1:2000); anti-slow MyHC (clone NOQ7.5.4D, Sigma-Aldrich, 1:2000); anti-pan-MyHC (clone MF20, R&D System Inc., USA, 1:4000); and anti-pan-actin (clone C4, Chemicon MAB 1501, 1:5000). Primary antibodies were diluted in Can Get Signal solution 1 (Toyobo, Japan). After washing three times in TBS-T for 10 min, membranes were incubated for 1 h with horseradish peroxidase-labeled rat anti-mouse IgG (Jackson ImmunoResearch, 415-036-166, USA) at a 1:5000 dilution with Can Get Signal solution 2 (Toyobo) and washed three times in TBS-T for 10 min. Bands were detected using enhanced chemiluminescence (ECL Western Blotting Reagents, RPN2106, GE Healthcare) and Hyperfilm ECL (GE Healthcare).

### 1.3 Results

**Fig. 1:**
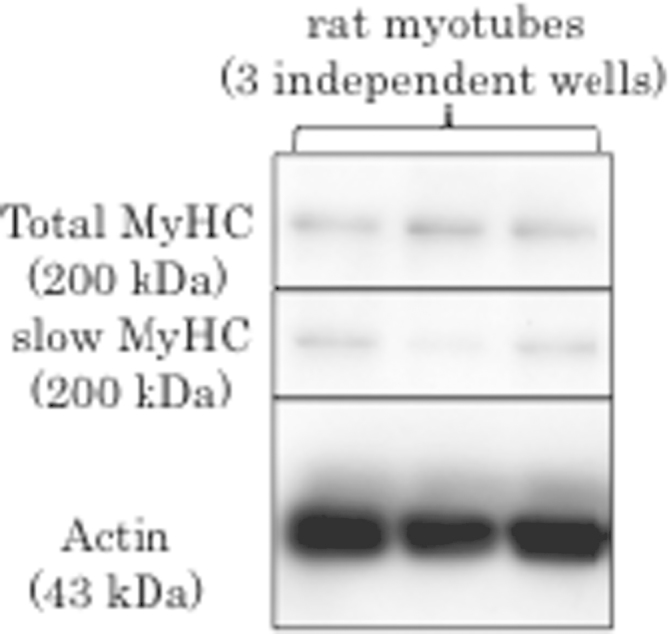
Detection of slow adult MyHC and total MyHC protein in myotubes differentiated for 6 days from cultures of primary satellite cells in Western blotting with specific antibodies. Three independent samples from different wells were used.

Previously, we found that immature neonatal and embryonic MyHC isoforms were dominant in myotubes differentiated from rat satellite cells [1]. The adult MyHC isoforms were not detected in silver-stained gels, which is a high-sensitivity, non-specific method of staining total protein. In this supporting study, we applied Western blotting using samples of myotubes differentiated from rat satellite cells in the relevant study [1]. We used three independent samples from different wells for reliability. All myotube samples contained a similarly high level of actin (detected with clone C4 antibody), indicating equal cell density and growth conditions in each well (Fig. 1, bottom panel). The obvious band for total MyHC (reaction with clone MF20 antibody) was easily detected (Fig. 1, upper panel). The slow adult isoform (reaction of clone NOQ7.5.4D antibody) was detected at low levels, in myotubes differentiated from rat satellite cells for 6 days (Fig. 1, middle panel). In these experimental conditions, there were no visible bands representing the presence of the fast adult isoform (probed using clone MY32 antibody). These findings suggest that in 6-day differentiated myotubes, adult MyHC isoforms were present, but at a very low level compared with the level of immature neonatal and embryonic MyHC isoforms, clearly detected in silver-stained gels [1]. We therefore, conclude that myotubes formed from rat satellite cells primarily express immature isoforms of MyHC, but contain a low but detectable amount of adult slow MyHC that can be detected by Western blotting. Thus, Western blotting may be a useful assay to evaluate muscle fiber type using primary myoblast cultures derived from isolated satellite cells, for example to test the effects of a growth factor that may regulate the development of fiber-type proportions during muscle regeneration.
